# Man's Superiority

**Published:** 1908-04-04

**Authors:** 


### EDITOR'S LETTER-BOX.

Our correspondents are reminded that prolixity is a great bar to publication, and that brevity of style and conciseness of statement greatly facilitate early insertion.

### MAN'S SUPERIORITY.

Sir,—Dr. Claye Shaw's clever lecture on "The Psychology of Woman," as noticed in your interesting annotation of March 28, will not make some of us forget that there has never been a female Shakespeare, Newton, or Michael Angelo. Most likely there never will be. Feminine and other disabilities did not prevent Joan of Arc from attaining distinction; but then, her's was not an intellectual field of achievement. Neither did they hinder more than one famous actress; but then, the histrionic art is the only one (why is this?) in which women have touched the highest levels. "Man's Superiority" seems something more than "boasted." I am, yours etc.,

London, March 28. X. Z.

